# 3,6-Dimethyl-1-phenyl-1*H*,4*H*-pyrano[2,3-*c*]pyrazol-4-one

**DOI:** 10.1107/S1600536812011348

**Published:** 2012-03-21

**Authors:** Abdullah M. Asiri, Hassan M. Faidallah, Salem A. Hameed, Seik Weng Ng, Edward R. T. Tiekink

**Affiliations:** a Chemistry Department, Faculty of Science, King Abdulaziz University, PO Box 80203, Jeddah, Saudi Arabia; b The Center of Excellence for Advanced Materials Research, King Abdulaziz University, Jeddah, PO Box 80203, Saudi Arabia; c Department of Chemistry, University of Malaya, 50603 Kuala Lumpur, Malaysia

## Abstract

The title compound, C_14_H_12_N_2_O_2_, is almost planar with an r.m.s. deviation for all non-H atoms of 0.038 Å. The observed planarity is rationalized in terms of a close intra­molecular C—H⋯O inter­action. Supra­molecular layers, two mol­ecules thick and with a step topology, are formed in the crystal packing *via* C—H⋯O contacts involving the carbonyl O atom, which accepts two such bonds, and π–π inter­actions between the components of the fused ring system and the phenyl ring of inversion-related mol­ecules [centroid–centroid distances = 3.6819 (13) and 3.6759 (12) Å].

## Related literature

For the analgesic and anti-inflammatory activities of pyrano[2,3-*c*]pyrazole derivatives, see: Kuo *et al.* (1984 ▶). For the synthesis, see: Gelin *et al.* (1983 ▶).
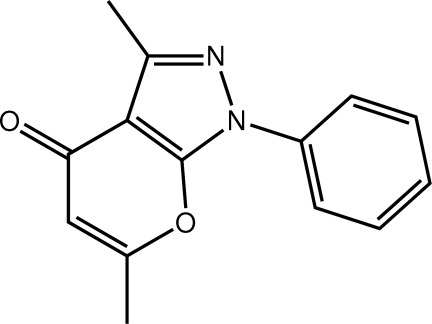


## Experimental

### 

#### Crystal data

C_14_H_12_N_2_O_2_
*M* *_r_* = 240.26
Triclinic, 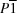

*a* = 6.7200 (6) Å
*b* = 8.2201 (8) Å
*c* = 11.2616 (7) Å
α = 93.914 (6)°
β = 95.162 (6)°
γ = 108.721 (8)°
*V* = 583.66 (9) Å^3^
*Z* = 2
Mo *K*α radiation
μ = 0.09 mm^−1^
*T* = 100 K
0.40 × 0.30 × 0.20 mm

#### Data collection

Agilent SuperNova Dual diffractometer with an Atlas detector
Absorption correction: multi-scan (*CrysAlis PRO*; Agilent, 2011 ▶) *T*_min_ = 0.964, *T*_max_ = 0.982
4356 measured reflections
2676 independent reflections
1946 reflections with *I* > 2σ(*I*)
*R*_int_ = 0.031

#### Refinement

*R*[*F* ^2^ > 2σ(*F* ^2^)] = 0.056
*wR*(*F* ^2^) = 0.158
*S* = 1.05
2676 reflections
165 parameters
H-atom parameters constrained
Δρ_max_ = 0.34 e Å^−3^
Δρ_min_ = −0.38 e Å^−3^

### 

Data collection: *CrysAlis PRO* (Agilent, 2011 ▶); cell refinement: *CrysAlis PRO*; data reduction: *CrysAlis PRO*; program(s) used to solve structure: *SHELXS97* (Sheldrick, 2008 ▶); program(s) used to refine structure: *SHELXL97* (Sheldrick, 2008 ▶); molecular graphics: *ORTEP-3* (Farrugia, 1997 ▶) and *DIAMOND* (Brandenburg, 2006 ▶); software used to prepare material for publication: *publCIF* (Westrip, 2010 ▶).

## Supplementary Material

Crystal structure: contains datablock(s) global, I. DOI: 10.1107/S1600536812011348/su2391sup1.cif

Structure factors: contains datablock(s) I. DOI: 10.1107/S1600536812011348/su2391Isup2.hkl

Supplementary material file. DOI: 10.1107/S1600536812011348/su2391Isup3.cml

Additional supplementary materials:  crystallographic information; 3D view; checkCIF report

## Figures and Tables

**Table 1:** Hydrogen-bond geometry (Å, °)

*D*—H⋯*A*	*D*—H	H⋯*A*	*D*⋯*A*	*D*—H⋯*A*
C10—H10⋯O1	0.95	2.33	2.970 (2)	124
C3—H3⋯O2^i^	0.95	2.47	3.400 (3)	167
C8—H8*C*⋯O2^ii^	0.98	2.54	3.472 (3)	158

Symmetry codes: (i) 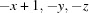; (ii) 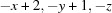.

**Fig. 1.**
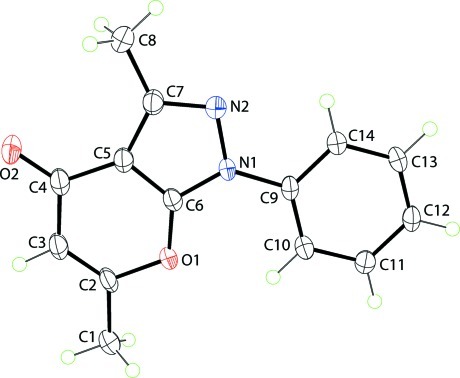
The molecular structure of the title molecule showing the atom-labelling. Displacement ellipsoids are drawn at the 50% probability level.

**Fig. 2.**
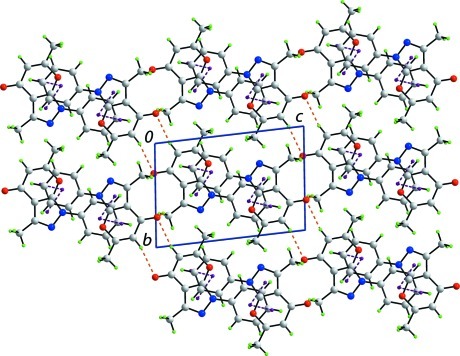
A view of the supramolecular layer in the bc plane in the crystal structure of the title compound. The O—H···O and π—π interactions are shown as orange and purple dashed lines, respectively.

**Fig. 3.**
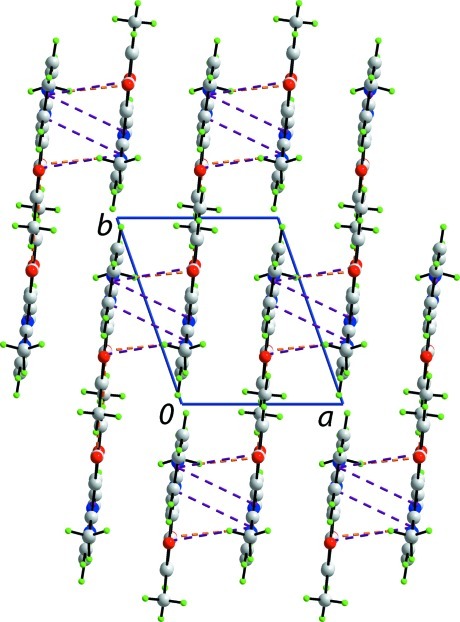
A view in projection down the c axis of the unit-cell contents of the title compound. The O—H···O and π—π interactions are shown as orange and purple dashed lines, respectively.

## supplementary crystallographic information

### Comment

It has been reported that many pyrano[2,3-*c*]pyrazole derivatives possess analgesic and anti-inflammatory activities (Kuo *et al.*, 1984). In this report, following literature precedents (Gelin *et al.*, 1983; Kuo *et al.*, 1984), the title compound was synthesized, and herein, its crystal and molecular structure are described.

In the title molecule, Fig.1, each of the pyrazole [r.m.s. deviation = 0.001 Å] and pyran-4-one [r.m.s. deviation = 0.006 Å] rings is planar and the dihedral angle between them is 0.82 (11)°. The planarity in the molecule extends to include the pendent phenyl ring, which makes a dihedral angle of 3.17 (11)° with the pyrazole ring. The r.m.s. deviation for the 18 non-hydrogen atoms is 0.038 Å, with maximum deviations of 0.071 (2) Å for atoms C13 and C14, and -0.059 (2) Å for the C10 atom. An explanation for the co-planarity in the molecule is the presence of intramolecular C10—H···O1 and C14—H14···N2 interactions (Table 1).

In the crystal packing, the carbonyl-O2 atom is bifurcated, forming two C—H···O interactions (Table 1 and Fig. 2), leading to a supramolecular layer in the *bc* plane. Layers are connected into double layers by π—π interactions involving the phenyl ring interacting with both rings of the fused ring system [ring centroid···ring centroid distances = 3.6819 (13) Å, for the five- and six-membered rings, and 3.6759 (12) Å, for the interaction between the two six-membered rings; symmetry operation: -*x+2*, -*y+1*, -*z+1*]. The layers have a step topology and stack along the *a* axis with no specific intermolecular interactions between them (Fig. 3).

### Experimental

Following literature precedents (Gelin *et al.*, 1983; Kuo *et al.*, 1984), dehydroacetic acid was converted to 4-acetoacetyl-3-methyl-1-phenyl-2-pyrazolin-5-one, which in turn yielded 3,6-dimethyl-1-phenyl-1*H*,3*aH*,4*H*,7*aH*-pyrano[2,3-*c*]pyrazol-4-one when treated with concentrated sulfuric acid.

To a solution of dehydroacetic acid (10 mmol) in benzene (20 ml) was added the phenylhydrazine (10 mmol). The mixture was refluxed for 30 min and allowed to stand at room temperature for 2 h. After the mixture was cooled, the hydrazone was collected and recrystallized from ethanol. A solution of this product (10 mmol) in acetic acid (20 ml) was refluxed for 1 h. After evaporation of the solvent, the residue was recrystallized from ethanol as needles. To a solution of this (2.5 g, 0.01 mmol), *i.e*. 4-acetoacetyl-3-methyl-1-phenyl-2-pyrazolin-5-one, in acetic acid (20 ml) was added concentrated sulfuric acid (1 ml) drop wise. The mixture was poured into cold water (150 ml) and the resulting precipitate was filtered, washed with 5% aqueous Na_2_CO_3_ solution, water, dried and recrystallized from ethanol. Yield: 74%. *M*.pt: 426–427 K.

### Refinement

Carbon-bound H-atoms were placed in calculated positions and were treated as riding atoms: C—H = 0.95 and 0.98 Å for CH and CH_3_ H atoms, respectively, with *U*_iso_(H) = k × *U*_eq_(C), where k = 1.5 for CH_3_ H atoms, and = 1.2 for other H atoms.

### Crystal data

**Table:**

C_14_H_12_N_2_O_2_	*Z* = 2
*M**_r_* = 240.26	*F*(000) = 252
Triclinic, *P*1	*D*_x_ = 1.367 Mg m^−^^3^
Hall symbol: -P 1	Mo *K*α radiation, λ = 0.71073 Å
*a* = 6.7200 (6) Å	Cell parameters from 1310 reflections
*b* = 8.2201 (8) Å	θ = 2.6–27.5°
*c* = 11.2616 (7) Å	µ = 0.09 mm^−^^1^
α = 93.914 (6)°	*T* = 100 K
β = 95.162 (6)°	Prism, orange
γ = 108.721 (8)°	0.40 × 0.30 × 0.20 mm
*V* = 583.66 (9) Å^3^	

### Data collection

**Table:**

Agilent SuperNova Dual diffractometer with an Atlas detector	2676 independent reflections
Radiation source: SuperNova (Mo) X-ray Source	1946 reflections with *I* > 2σ(*I*)
Mirror monochromator	*R*_int_ = 0.031
Detector resolution: 10.4041 pixels mm^-1^	θ_max_ = 27.6°, θ_min_ = 2.6°
ω scan	*h* = −8→8
Absorption correction: multi-scan (*CrysAlis PRO*; Agilent, 2011)	*k* = −10→9
*T*_min_ = 0.964, *T*_max_ = 0.982	*l* = −14→14
4356 measured reflections	

### Refinement

**Table:**

Refinement on *F*^2^	Primary atom site location: structure-invariant direct methods
Least-squares matrix: full	Secondary atom site location: difference Fourier map
*R*[*F*^2^ > 2σ(*F*^2^)] = 0.056	Hydrogen site location: inferred from neighbouring sites
*wR*(*F*^2^) = 0.158	H-atom parameters constrained
*S* = 1.05	*w* = 1/[σ^2^(*F*_o_^2^) + (0.0761*P*)^2^ + 0.084*P*] where *P* = (*F*_o_^2^ + 2*F*_c_^2^)/3
2676 reflections	(Δ/σ)_max_ = 0.001
165 parameters	Δρ_max_ = 0.34 e Å^−^^3^
0 restraints	Δρ_min_ = −0.38 e Å^−^^3^

### Fractional atomic coordinates and isotropic or equivalent isotropic displacement parameters (Å^2^)

**Table:**

	*x*	*y*	*z*	*U*_iso_*/*U*_eq_	
O1	0.6182 (2)	0.25349 (16)	0.35598 (11)	0.0238 (3)	
O2	0.6446 (2)	0.28672 (19)	−0.00902 (12)	0.0349 (4)	
N1	0.7795 (2)	0.55863 (19)	0.37569 (13)	0.0216 (4)	
N2	0.8481 (3)	0.6876 (2)	0.30000 (14)	0.0257 (4)	
C1	0.4637 (3)	−0.0519 (3)	0.33142 (18)	0.0336 (5)	
H1A	0.4058	−0.1514	0.2706	0.050*	
H1B	0.3523	−0.0428	0.3793	0.050*	
H1C	0.5797	−0.0671	0.3838	0.050*	
C2	0.5447 (3)	0.1084 (2)	0.27152 (17)	0.0266 (4)	
C3	0.5520 (3)	0.1187 (3)	0.15402 (17)	0.0284 (5)	
H3	0.4976	0.0145	0.1020	0.034*	
C4	0.6380 (3)	0.2797 (3)	0.10026 (17)	0.0276 (5)	
C5	0.7110 (3)	0.4278 (2)	0.18953 (16)	0.0245 (4)	
C6	0.6979 (3)	0.4044 (2)	0.30916 (16)	0.0221 (4)	
C7	0.8072 (3)	0.6086 (3)	0.18951 (17)	0.0266 (4)	
C8	0.8643 (4)	0.7086 (3)	0.08486 (18)	0.0339 (5)	
H8A	0.9106	0.8324	0.1112	0.051*	
H8B	0.7406	0.6789	0.0243	0.051*	
H8C	0.9792	0.6801	0.0502	0.051*	
C9	0.8063 (3)	0.6061 (2)	0.50196 (16)	0.0221 (4)	
C10	0.7333 (3)	0.4863 (2)	0.58291 (17)	0.0262 (4)	
H10	0.6643	0.3678	0.5554	0.031*	
C11	0.7627 (3)	0.5423 (3)	0.70503 (17)	0.0261 (4)	
H11	0.7132	0.4613	0.7609	0.031*	
C12	0.8638 (3)	0.7155 (3)	0.74549 (17)	0.0262 (4)	
H12	0.8826	0.7529	0.8287	0.031*	
C13	0.9367 (3)	0.8327 (3)	0.66445 (17)	0.0269 (4)	
H13	1.0062	0.9511	0.6922	0.032*	
C14	0.9096 (3)	0.7798 (2)	0.54306 (17)	0.0259 (4)	
H14	0.9611	0.8613	0.4878	0.031*	

### Atomic displacement parameters (Å^2^)

**Table:**

	*U*^11^	*U*^22^	*U*^33^	*U*^12^	*U*^13^	*U*^23^
O1	0.0281 (7)	0.0183 (7)	0.0214 (7)	0.0041 (5)	0.0027 (5)	−0.0041 (5)
O2	0.0417 (9)	0.0379 (9)	0.0209 (7)	0.0093 (7)	0.0037 (6)	−0.0068 (6)
N1	0.0251 (8)	0.0193 (8)	0.0177 (8)	0.0049 (6)	0.0014 (6)	−0.0039 (6)
N2	0.0310 (9)	0.0230 (8)	0.0214 (8)	0.0064 (7)	0.0042 (6)	0.0004 (7)
C1	0.0416 (13)	0.0218 (11)	0.0314 (11)	0.0035 (9)	0.0046 (9)	−0.0050 (9)
C2	0.0272 (10)	0.0194 (10)	0.0277 (10)	0.0035 (8)	0.0003 (8)	−0.0092 (8)
C3	0.0299 (11)	0.0264 (11)	0.0255 (10)	0.0075 (8)	0.0007 (8)	−0.0086 (8)
C4	0.0263 (10)	0.0290 (11)	0.0241 (10)	0.0074 (8)	−0.0002 (8)	−0.0073 (8)
C5	0.0253 (10)	0.0253 (10)	0.0214 (10)	0.0079 (8)	0.0019 (7)	−0.0033 (8)
C6	0.0213 (9)	0.0201 (9)	0.0232 (9)	0.0061 (7)	0.0014 (7)	−0.0036 (7)
C7	0.0280 (10)	0.0278 (10)	0.0227 (10)	0.0083 (8)	0.0037 (7)	−0.0023 (8)
C8	0.0439 (13)	0.0326 (12)	0.0229 (10)	0.0091 (10)	0.0066 (9)	0.0014 (9)
C9	0.0229 (9)	0.0237 (10)	0.0183 (9)	0.0079 (8)	−0.0005 (7)	−0.0049 (7)
C10	0.0295 (10)	0.0215 (10)	0.0243 (10)	0.0057 (8)	0.0018 (8)	−0.0048 (8)
C11	0.0309 (11)	0.0252 (10)	0.0220 (10)	0.0089 (8)	0.0042 (8)	0.0007 (8)
C12	0.0291 (10)	0.0283 (11)	0.0194 (9)	0.0092 (8)	0.0003 (7)	−0.0060 (8)
C13	0.0302 (10)	0.0216 (10)	0.0241 (10)	0.0047 (8)	−0.0014 (8)	−0.0069 (8)
C14	0.0306 (11)	0.0227 (10)	0.0215 (10)	0.0055 (8)	0.0033 (8)	−0.0019 (8)

### Geometric parameters (Å, º)

**Table:**

O1—C6	1.348 (2)	C5—C7	1.418 (3)
O1—C2	1.397 (2)	C7—C8	1.491 (3)
O2—C4	1.240 (2)	C8—H8A	0.9800
N1—C6	1.349 (2)	C8—H8B	0.9800
N1—N2	1.394 (2)	C8—H8C	0.9800
N1—C9	1.428 (2)	C9—C10	1.391 (3)
N2—C7	1.326 (2)	C9—C14	1.396 (3)
C1—C2	1.489 (3)	C10—C11	1.396 (3)
C1—H1A	0.9800	C10—H10	0.9500
C1—H1B	0.9800	C11—C12	1.390 (3)
C1—H1C	0.9800	C11—H11	0.9500
C2—C3	1.336 (3)	C12—C13	1.378 (3)
C3—C4	1.460 (3)	C12—H12	0.9500
C3—H3	0.9500	C13—C14	1.384 (2)
C4—C5	1.447 (3)	C13—H13	0.9500
C5—C6	1.380 (3)	C14—H14	0.9500
			
C6—O1—C2	114.47 (15)	N2—C7—C8	120.75 (18)
C6—N1—N2	109.07 (14)	C5—C7—C8	128.11 (17)
C6—N1—C9	132.12 (16)	C7—C8—H8A	109.5
N2—N1—C9	118.81 (14)	C7—C8—H8B	109.5
C7—N2—N1	106.27 (15)	H8A—C8—H8B	109.5
C2—C1—H1A	109.5	C7—C8—H8C	109.5
C2—C1—H1B	109.5	H8A—C8—H8C	109.5
H1A—C1—H1B	109.5	H8B—C8—H8C	109.5
C2—C1—H1C	109.5	C10—C9—C14	120.13 (17)
H1A—C1—H1C	109.5	C10—C9—N1	122.27 (16)
H1B—C1—H1C	109.5	C14—C9—N1	117.61 (17)
C3—C2—O1	122.72 (18)	C9—C10—C11	119.17 (18)
C3—C2—C1	126.60 (18)	C9—C10—H10	120.4
O1—C2—C1	110.67 (17)	C11—C10—H10	120.4
C2—C3—C4	124.29 (18)	C12—C11—C10	120.53 (19)
C2—C3—H3	117.9	C12—C11—H11	119.7
C4—C3—H3	117.9	C10—C11—H11	119.7
O2—C4—C5	124.75 (19)	C13—C12—C11	119.72 (18)
O2—C4—C3	123.43 (18)	C13—C12—H12	120.1
C5—C4—C3	111.82 (17)	C11—C12—H12	120.1
C6—C5—C7	104.07 (16)	C12—C13—C14	120.65 (18)
C6—C5—C4	119.77 (18)	C12—C13—H13	119.7
C7—C5—C4	136.14 (18)	C14—C13—H13	119.7
N1—C6—O1	123.63 (16)	C13—C14—C9	119.81 (18)
N1—C6—C5	109.45 (17)	C13—C14—H14	120.1
O1—C6—C5	126.92 (17)	C9—C14—H14	120.1
N2—C7—C5	111.14 (17)		
			
C6—N1—N2—C7	−0.21 (19)	C7—C5—C6—O1	−179.70 (17)
C9—N1—N2—C7	179.19 (15)	C4—C5—C6—O1	−1.3 (3)
C6—O1—C2—C3	−0.3 (3)	N1—N2—C7—C5	0.2 (2)
C6—O1—C2—C1	178.93 (15)	N1—N2—C7—C8	−178.94 (17)
O1—C2—C3—C4	0.7 (3)	C6—C5—C7—N2	−0.2 (2)
C1—C2—C3—C4	−178.48 (18)	C4—C5—C7—N2	−178.2 (2)
C2—C3—C4—O2	179.56 (19)	C6—C5—C7—C8	178.93 (19)
C2—C3—C4—C5	−1.1 (3)	C4—C5—C7—C8	0.9 (4)
O2—C4—C5—C6	−179.34 (18)	C6—N1—C9—C10	−3.8 (3)
C3—C4—C5—C6	1.4 (2)	N2—N1—C9—C10	176.97 (16)
O2—C4—C5—C7	−1.6 (4)	C6—N1—C9—C14	176.32 (19)
C3—C4—C5—C7	179.1 (2)	N2—N1—C9—C14	−2.9 (2)
N2—N1—C6—O1	179.85 (15)	C14—C9—C10—C11	0.7 (3)
C9—N1—C6—O1	0.6 (3)	N1—C9—C10—C11	−179.21 (16)
N2—N1—C6—C5	0.1 (2)	C9—C10—C11—C12	−0.1 (3)
C9—N1—C6—C5	−179.18 (17)	C10—C11—C12—C13	−0.4 (3)
C2—O1—C6—N1	−179.01 (16)	C11—C12—C13—C14	0.2 (3)
C2—O1—C6—C5	0.7 (3)	C12—C13—C14—C9	0.4 (3)
C7—C5—C6—N1	0.0 (2)	C10—C9—C14—C13	−0.8 (3)
C4—C5—C6—N1	178.43 (16)	N1—C9—C14—C13	179.05 (15)

### Hydrogen-bond geometry (Å, º)

**Table:**

*D*—H···*A*	*D*—H	H···*A*	*D*···*A*	*D*—H···*A*
C10—H10···O1	0.95	2.33	2.970 (2)	124
C14—H14···N2	0.95	2.39	2.748 (2)	102
C3—H3···O2^i^	0.95	2.47	3.400 (3)	167
C8—H8*C*···O2^ii^	0.98	2.54	3.472 (3)	158

Symmetry codes: (i) −*x*+1, −*y*, −*z*; (ii) −*x*+2, −*y*+1, −*z*.
